# High intestinal parasite infection detected in children from Región Autónoma Atlántico Norte (R.A.A.N.) of Nicaragua

**DOI:** 10.1038/s41598-022-09756-y

**Published:** 2022-04-07

**Authors:** Carla Muñoz-Antoli, Paloma Pérez, Aleyda Pavón, Rafael Toledo, José Guillermo Esteban

**Affiliations:** 1 Área Parasitología, Departamento Farmacia y Tecnología Farmacéutica y Parasitología, Facultad Farmacia, Universidad Valencia, Valencia, Spain; 2 Departamento Bioanálisis Clínico, Instituto Politécnico de La Salud (IPS-Polisal), Universidad Nacional Autónoma de Nicaragua, Managua, Nicaragua

**Keywords:** Diseases, Risk factors

## Abstract

There is a lack of epidemiological information concerning intestinal parasitic infections, and especially in soil-transmitted helminths, occurring in some departments of Nicaragua. Up to now, this is the first study involving two nearby areas (Puerto Cabezas and Siuna municipalities) of the Región Autónoma Atlántico Norte (R.A.A.N.). One stool sample was analyzed by Kato-Katz, formaldehyde-ethyl acetate concentration method and modified Ziehl–Neelsen technique, and a simple questionnaire concerning demographic, sanitary and behavioral data was distributed among 735 children and evaluated. Overall prevalence of infection reached 97.0%, being the highest prevalences detected in all Nicaragua. The higher protozoan prevalence appears in Siuna (94.5%), a rural interior municipality, with a typical tropical monsoon climate, while the higher helminths rates were reached in Puerto Cabezas (92.8%), the urbanized coastal capital, with a typical tropical rainforest climate. No statistical differences were found with regard to sex. However, the 6–11-year age-group children presented the highest prevalences. Most *T. trichiura* infections (59.4%) were of light intensity, while 51.7% of *Ascaris lumbricoides* were of moderate intensity. Multivariable logistic regression analysis indicated that those who drink rainwater and walk barefoot were 2.9 and 2.5 times more likely to have helminth infections, respectively. Results from one geographical setting might not be applied to other nearby with different climatic conditions. The use of anthelmintic drugs only will not be sufficient to bring prevalence to low levels. It is necessary to design geographically more specific intervention, with communication and interaction between different disciplines (e.g. parasitology, biochemistry, molecular biology, epidemiology, public health, etc.) being imperative to reduce STH infection.

## Introduction

World Health Organization (WHO) list of Neglected Tropical Diseases (NTD) include several intestinal parasitic infections (IPI) that are most prevalent in developing countries where high burdens of morbidity and mortality appear^1,2^.

According to WHO data, a large part of the world population, basically school-age children are infected with a broad spectrum of parasitic protozoa and helminths^3^.

Poor socio-economic and sanitation situations are related to protozoan infections, that even cause morbidity problems when they reach mild intensity and/or concomitant infections^4–7^.

Among helminths, soil-transmitted helminths (STH) are among the most common infections worldwide, with a large proportion occurring in developing countries of the Americas, in China and East Asia, as well as Sub-Saharan Africa^8^.

IPI in children can lead to severe complications such as malabsorption, malnutrition, growth- and development disorders, anemia, diarrhea as well as physical and mental consequences constituting important health and social problems^9^.

Differences in the prevalence rates of intestinal parasites can be associated with environmental factors, such as vegetation, temperature, humidity and precipitation, as well as geographical site, and a variety of cultural, economic, and social characteristics^10,11^. All of this is linked to the lack of access to safe water and sanitation, poor hygiene practices and unsafe human waste disposal^12^.

Nicaragua is the largest country of Central America, with approximately 6 million inhabitants, occupying a landmass of 130,967 km2, and being the second poorest country in the Latin American and Caribbean (LAC) region. This country is made up of three regions: the Pacific region, with seven departments; the Center region, with eight departments; and the Atlantic/Caribbean region including the Región Autónoma Atlántico Norte (R.A.A.N.) and the Región Autónoma Atlántico Sur (R.A.A.S.). In the 1990s, Nicaragua established massive deworming programs, with a joint effort of various actors (ministries, NGOs, companies, etc.) in such a way that an 87% coverage of the child population was reported in 2011, and nowadays deworming is included as a new category on child vaccination cards^13^.

Several studies have been conducted on the prevalence of intestinal parasitic infections in Nicaragua^14–21^, although this is the first study published in SCI journals involving R.A.A.N. The goal of this epidemiological study was to determine the prevalence of intestinal parasite infections and the intensity of STH, assessing which variables (demographic/sanitary/behavioral) among schoolchildren were related to the infection, providing the baseline for appropriate strategies against STH infections.

## Methods

### Study area and climatic conditions

**Figure 1:**
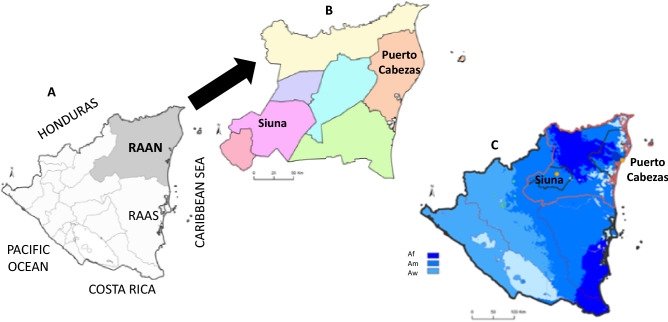
(**A**) Map of the study area Región Autónoma Atlántico Norte (R.A.A.N.) in Nicaragua; (**B**) Detail of municipalities of R.A.A.N.; (**C**) Different climatology conditions: Tropical rainforest climate (Af) in Puerto Cabezas; Tropical monsoon climate (Am) in Siuna; and tropical savanna climate with dry-winter characteristics (Aw) (according to Köppen-Geiger climate classification). We use administrative area spatial data from the Global Administrative Area Database (GADM) v3.6 and the Köppen-Geiger climate classification system at 1 km resolution for the present (1980-2016) (Beck et al. 2018: 10.1038/sdata.2018.214) using QGIS software v3.22.5 (https://www.qgis.org/en/site/) to represent the study area and its climate classification.

R.A.A.N. covers the largest area (33,105 km^2^) of Nicaragua, covering 26.3% of the national territory (Fig. 1A). It borders Honduras to the north and has a long coastline along the Caribbean Sea. R.A.A.N., with an estimated population of around 490,390 inhabitants, is divided into eight municipalities (Fig. 1B). The administrative capital is the municipality of Puerto Cabezas (lat. 14°03′N, long. 83°22′W) with around 113,534 inhabitants and 63% from urban areas. The second largest municipality is Siuna (lat. 13°44′N, long. 84°46′W), with around 103,139 inhabitants and 84% belonging to rural areas. In general, homes in rural areas of both municipalities tend to be more dispersed, being surrounded by grassland, without any urbanization and without sanitation.

According to the Köppen-Geiger climate classification^22^, the Pacific region in Nicaragua shows a tropical savanna climate (with a pronounced dry season while the Atlantic region have: a) a tropical monsoon climate (Am), with 2400 to 3000 mm annual rainfall, temperature oscillating between 24 °C and 29 °C, with a winter or rainy season (from May to January) and a summer or dry season (from February to April) with only sporadic rains, typical conditions of the municipality of Siuna; and b) a tropical rainforest climate (Af), with 2000 to 6000 mm annual rainfall, temperature oscillating between 27 °C and 39 °C, 80%-90% relative humidity, with a rainy season only (Fig. 1C), typical conditions of the municipality of Puerto Cabezas.

### Population and sample collection

The survey was carried out in both municipalities, Puerto Cabezas and Siuna, involving randomly selected schools. The school teachers and the students’ parents were informed about the objective of the study, allowing schoolchildren and their little siblings to participate voluntarily. The sample size (about 25,000 children, based on a level of confidence of 95%, an expected prevalence of 50%, and a marginal error of 5%) was estimated in 378 children.

The survey was carried out from February to April 2015 and finally involved a total of 735 children (342 boys and 393 girls) aged 2–15 years (mean ± S.D. = 10 ± 1.2) coming from both municipalities: 318 from Puerto Cabezas (185 from an urban school and 133 from a rural school), and 417 from Siuna (140 from an urban school and 277 from a rural school). In both municipalities, urban schools were determined with paved ground and cobbled streets while rural schools by no urbanization and surrounded by grassland. A clean, plastic, numbered container with a snap-on lid was handed out to each child. With the aid of parents/teachers, if necessary, one stool sample per child was collected and a simple questionnaire concerning various data of the children (sex, age and area; water supply; sanitation; habits) was filled out.

### Laboratory methods

A Kato-Katz slide was made from each stool sample and examined, within 30–60 min after preparation, to count the number of eggs of STHs. The number of eggs per gram of feces (EPG) was calculated by multiplying by 24 the total egg counts in all fields. WHO criteria (reference range values for each STH species) were used to classify each infection as light, moderate or heavy intensity infections^23^.

The rest of the fecal material of each sample was fixed in 10% formalin solution (1:3) and shipped to the Institute Politécnico de la Salud (IPS) laboratory in Managua where all samples were concentrated using the formaldehyde-ethyl acetate concentration technique (FECT). Finally, each fecal sample was microscopically examined at Department of Parasitology, Universidad de Valencia (Spain), and one aliquot of sediment obtained with FECT was stained using a modified Ziehl–Neelsen technique (MZNT). The FECT and the Kato-Katz slides were used to obtain prevalence data of STHs.

### Data analysis

Statistical analyses were carried out using the SPSS version 19 software package for Windows (SPSS Inc, Chicago, IL, USA). Statistical comparison of categorical variables was carried out with the Chi-squared test. Univariable analysis, OR (95%b CI) and significance levels of all independent variables were assessed. Factors identified as statistically significant at the 5% level in univariable analysis were entered into a multiple logistic regression model. All results were considered significant if the *p* value was < 0.05.

### Ethical approval and consent to participate

Universidad de Valencia. Estudi General granted the ethical approval of the study (H1477378643204) in accordance with the Declaration of Helsinki. Verbal informed consent was obtained from the parents/guardians of children enrolled. Diagnostic results were sent to IPS which informed the Nicaraguan Ministry of Health, being in charge of appropriate treatments.

### Consent for publication

Verbal consent for children publication was obtained from the parents/guardians.

## Results

### Total infections

Table 1 summarizes the total infection results obtained in R.A.A.N., stratified by municipalities. The entire spectrum was made up to 10 protozoa and 6 helminth species, although not all parasite species were found in both municipalities. The isolated cases of *Balantidium coli* detected in Puerto Cabezas municipality and of *Cryptosporidium* sp., *Taenia* sp. *and Strongyloides stercoralis* detected in Siuna municipality are noteworthy.

**Table 1:** Results of the total parasite spectrum and prevalence rates obtained in the two municipalities and in the total study in Región Autónoma Atlántico Norte (R.A.A.N.) of Nicaragua.

	Puerto Cabezas N^c^ = 318		Siuna N = 417		R.A.A.N N = 735	
	n^d^	% (95%CI^e^)	n	% (95%CI)	n	% (95%CI)
**Protozoa**	**273**	**85.8 (81.7–89.3)**	**394**	**94.5 (92.0–96.4)**	**667**	**90.7 (88.5–92.7)**
*Entamoeba coli*	158	49.7 (44.2–55.2)	148	35.5 (31.0–40.2)	306	41.6 (38.1–45.2)
*Entamoeba* complex^a^	41	12.9 (9.6–16.9)	75	18.0 (14.5–21.9)	116	15.8 (13.3–18.6)
*Entamoeba hartmanni*	29	9.1 (6.3–12.7)	100	24.0 (20.1–28.2)	129	17.6 (14.9–20.4)
*Endolimax nana*	93	29.2 (24.5–34.4)	163	39.1 (34.5–43.8)	256	34.8 (31.5–38.3)
*Iodamoeba bütschlii*	14	4.4 (2.5–7.1)	35	8.4 (6.0–11.3)	49	3.0 (1.9–4.4)
*Chilomastix mesnili*	4	1.3 (0.4–3.0)	18	4.3 (2.7–6.6)	22	2.5 (1.4–3.6)
*Giardia intestinalis*	143	45.0 (39.6–50.5)	190	45.6 (40.8–50.4)	333	45.3 (41.7–48.9)
*Cryptosporidium* spp.	0	0	1	0.2 (0–1.1)	1	0.1 (0–0.6)
*Balantidium coli*	1	0.3 (0–0.9)	0	0	1	0.1 (0–0.3)
*Blastocystis* spp.	217	68.2 (63.0–73.2)	334	80.1 (76.1–83.7)	551	75.0 (71.7–78.0)
**Helminths**	**295**	**92.8 (89.5–95.2)**	**158**	**37.9 (33.3–42.6)**	**453**	**61.6 (58.1–65.1)**
*Hymenolepis nana*	27	8.5 (5.8–11.9)	4	1 (0.3–2.3)	31	4.2 (2.9–5.9)
*Taenia sp.*	0	0	1	0.2 (0–0.6)	1	0.1 (0–0.3)
*Trichuris trichiura*	280	88.1 (84.1–91.3)	134	32.1 (27.8–36.7)	414	56.3 (52.7–59.8)
*Ascaris lumbricoides*	212	66.7 (61.4–71.7)	59	14.1 (11.1–17.7)	271	36.9 (33.4–40.4)
Hookworm^b^	27	8.5 (5.8–11.9)	46	11.0 (8.3–14.3)	73	9.9 (7.9–12.3)
*Strongyloides stercoralis*	0	0	3	0.7 (0–1.5)	3	0.4 (2.7–5.7)
**Total**	**316**	**99.4 (98.0–99.9)**	**397**	**95.2 (92.8–97.0)**	**713**	**97.0 (95.6–98.1)**

^a^*Entamoeba histolytica/E. dispar/E. moshkovskii.*

^b^*Ancylostoma duodenale* and/or *Necator americanus.*

^c^Number of schoolchildren studied.

^d^Number of schoolchildren parasitized.

^e^95% confidence interval.

Significance values are given in bold.

Overall prevalence of infection, with at least one parasite species, reached 97.0% in the children surveyed, either being protozoa (90.7%) or helminths (61.6%). Among the R.A.A.N. children surveyed, protozoan infections were more prevalent than helminths, with statistical differences (*p* < 0.0001).

*Blastocystis* spp. (75.0%) was the most common intestinal protozoan diagnosed, followed by *Giardia intestinalis* (45.3%). Among STH, *Trichuris trichiura*,* Ascaris lumbricoides* and Hookworm were the most prevalent ones (56.3%, 36.9% and 9.9%, respectively).

The overall prevalence of infection presents statistical significant differences (*p* = 0.002) among both municipalities studied (99.4% in Puerto Cabezas vs 95.2% in Siuna), with a higher protozoan prevalence in Siuna municipality (94.5%) (*p* < 0.0001), whereas a higher helminth prevalence was reached in Puerto Cabezas (92.8%) (*p* < 0.0001). Among STH, *T. trichiura* and *A. lumbricoides* prevalences were higher in Puerto Cabezas than in Siuna, with statistically significant differences (*p* < 0.0001). In Hookworm, although higher prevalence rates occur in Siuna (11.0%), no significant differences were detected (*p* = 0.188).

### Gender, age groups and urban/rural area infections

Prevalence results according to gender, age groups and urban/rural school area are shown in Table 2. No statistical differences were found with regard to gender neither in the total prevalence rates nor within each municipality.

**Table 2:** Prevalence results of protozoa, helminths, and total infection according to gender, age group and urban/rural school area obtained in both municipalities studied and in the total R.A.A.N. (Nicaragua).

	Puerto Cabezas			Siuna			R.A.A.N		
	Protozoa	Helminths	Total	Protozoa	Helminths	Total	Protozoa	Helminths	Total
	%^a^ (n/N)^b^	% (n/N)	% (n/N)	% (n/N)	% (n/N)	% (n/N)	% (n/N)	% (n/N)	% (n/N)
**Gender**									
Female	87 (160/184)	91.8 (169/184)	98.9 (182/184)	94.7 (198/209)	37.3 (78/209)	94.7 (198/209)	91.1 (358/393)	62.8 (247/393)	96.7 (380/393)
Male	84.3 (113/134)	94.0 (126/134)	100 (134/134)	94.2 (196/208)	38.5 (80/208)	95.7 (199/208)	90.4 (309/342)	60.2 (206/342)	97.4 (333/342)
**Age group (years)**									
2–5	92.3 (48/52)	90.3 (47/52)	98.0 (51/52)	90.4 (142/157)	33.1 (52/157)	92.4 (145/157)	90.9 (190/209)	47.4 (99/209)	93.3 (195/209)
6–11	86.4 (184/213)	94.8 (202/213)	100 (213/213)	96.8 (183/189)	43.4 (82/189)	96.8 (183/189)	91.3 (367/402)	70.6 (284/402)	99.0 (398/402)
12–15	77.4 (41/53)	86.8 (46/53)	98.1 (52/53)	97.2 (69/71)	33.8 (24/71)	97.2 (69/71)	88.7 (110/124)	56.5 (70/124)	96.8 (120/124)
**School area**									
Urban	87.6 (162/185)	93.0 (172/185)	99.5 (184/185)	95.7 (134/140)	37.1 (52/140)	96.4 (135/140)	91.1 (296/325)	68.9 (224/325)	98.2 (319/325)
Rural	83.5 (111/133)	92.5 (123/133)	99.2 (132/133)	93.9 (260/277)	38.3 (106/277)	94.6 (262/277)	90.5 (371/410)	55.9 (229/410)	96.1 (394/410)

^a^Percentage.

^b^Positive students/total students in the group.

The distribution of total infection prevalence according to age groups revealed significant differences. Total prevalence increased, always being highest in the group of 6–11 years-old children, both in Puerto Cabezas (*p* = 0.006) and Siuna (*p* = 0.036) municipalities. However, protozoa infection in Siuna municipality was higher in the adolescent group with statistical differences (*p* = 0.019) and it was higher in the municipality of Puerto Cabezas in the youngest age group with statistical differences (*p* = 0.049).

The total infection rate of helminths in urban schools was significantly higher than in rural schools (*p* = 0.0003). In particular, *A. lumbricoides* prevalence rates obtained in urban schools were higher than those obtained in rural schools (*p* < 0.0001), while Hookworm prevalence rates were higher in rural schools (*p* = 0.005). No differences between the two areas were detected in the case of *T. trichiura* infections (*p* = 0.367), with similar rates in urban (49.7%) as well as rural (46.3%) schools.

### Polyparasitism

Most infected children presented polyparasitism (86.9%) with statistical differences (*p* < 0.0001) to monoinfection. This monoinfection resulted more frequent in Siuna (11.6%) than in Puerto Cabezas, with statistical differences (*p* = 0.007).

The highest percentages correspond to co-infection with three (21.3%), two (19.2%) and four (18.5%) different species, although children harboring five to ten species were also detected (from 13.9 to 0.1%, respectively). Only one 8-year-old boy from Siuna municipality harbored 10 species: *E. coli*,* E. hartmanni*,* E. nana*,* I. buetschlii*,* G. intestinalis*,* Blastocystis* spp., *T. trichiura*,* A. lumbricoides*, hookworm and *S. stercoralis*.

**Figure 2:**
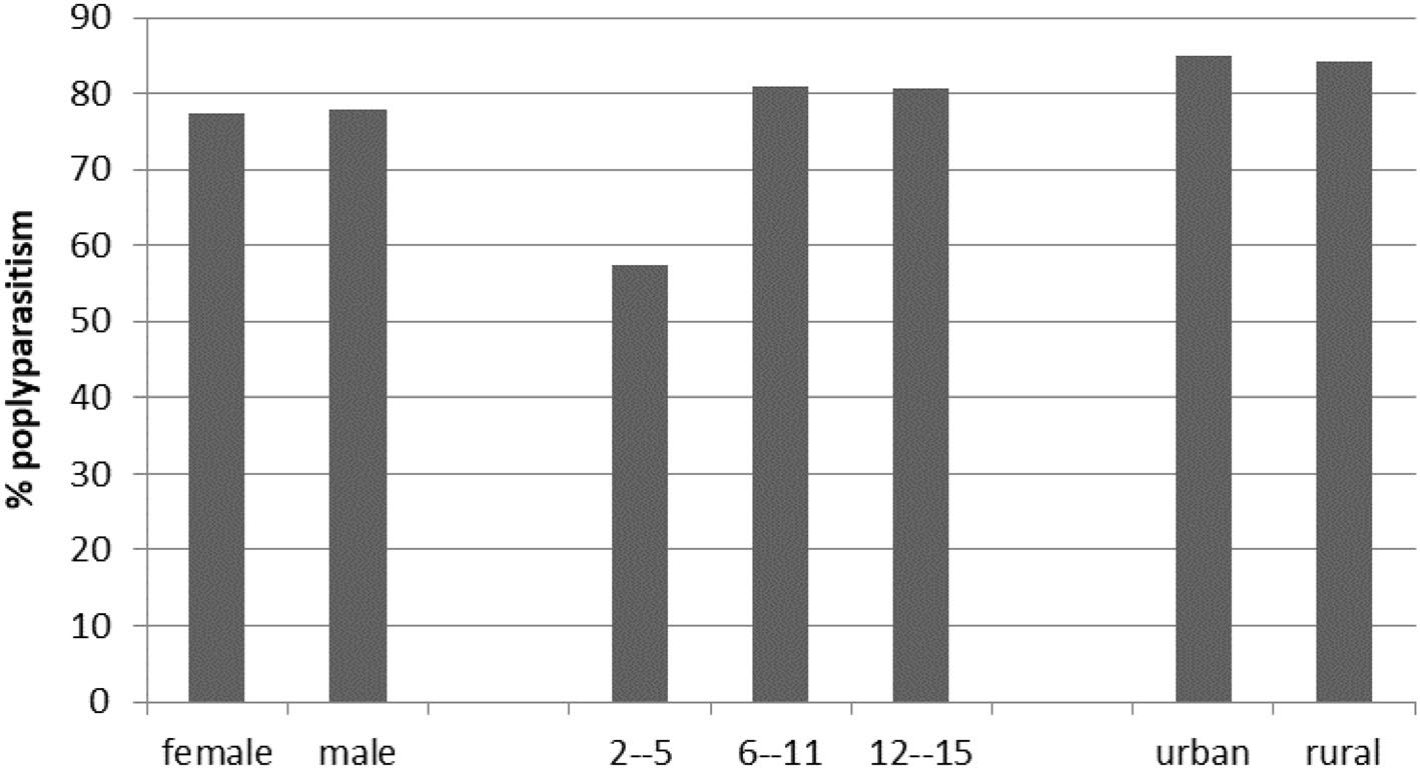
Polyparasitism obtained in R.A.A.N. according to sex, age group and urban/rural school area.

The extent of polyparasitism in the entire study according to gender, age groups and urban/rural school area is shown in Fig. 2. There were no statistical differences between females and males or between urban and rural schools. However, polyparasitism was lower (57.3%), with statistical differences (*p* < 0.0001), among the youngest children studied.

### STH infection intensity and grouping

*T. trichiura*,* A. lumbricoides* and Hookworm median (range) infection intensity in R.A.A.N. was 960 (24–88.752), 18.096 (24–149.544) and 24 (24–264) eggs per gram (epg) of feces, respectively (Table 3).

**Table 3:** STH infection intensity (eggs per gram of feces) obtained in the two municipalities studied and in the total R.A.A.N. (Nicaragua).

	**Puerto Cabezas**	**Siuna**	**R.A.A.N**
	N^a^ = 318	N = 417	N = 735
***T. trichiura***			
Range	24–88,752	24–13,248	24–88,752
AM^b^	3659.7	4452	4055.8
GM^c^	1021.5	2870.4	1078.3
***A. lumbricoides***			
Range	24–149,544	24–54,480	24–149,544
AM	29,612.2	13,778.7	21,695.4
GM	11,768.1	3676.3	7727.9
**Hookworm**			
Range	24–264	24–264	24–264
AM	190.1	144	158.8
GM	154.9	117.8	128.6

^a^Number of schoolchildren studied.

^b^Arithmetic mean of eggs per gram of feces.

^c^Geometric mean of eggs per gram of feces.

Table 4 shows the prevalence of each class of infection intensity obtained in the two municipalities, according to gender, age group and urban/rural schools studied. Among those infected, most *T. trichiura* infections were light intensity (59.4%), while 51.7% of *A. lumbricoides* were moderate-intensity infections. No differences in infection intensity classes appeared among the two municipalities studied in any STH species.

**Table 4:** Percentage of each class of infection intensity of STH species according to gender, age group and school area obtained in both municipalities studied and in the total R.A.A.N. (Nicaragua).

	Puerto Cabezas	Siuna	R.A.A.N	Gender		Age group (years)			School area	
				Female	Male	2–5	6–11	12–15	Urban	Rural
***T. trichiura***										
Light n/N^a^	172/280	74/134	246/414	147/222	99/192	36/58	166/284	44/72	125/187	121/227
%^b^	61.4	55.2	59.4	66.2	51.6	62.1	58.5	61.1	66.8	53.3
Moderate n/N	89/280	49/134	138/414	74/222	64/192	23/58	96/284	19/72	58/187	80/227
%	31.8	36.6	33.3	33.3	33.3	39.7	33.8	26.4	31.0	35.2
Heavy n/N	19/280	11/134	30/414	11/222	19/192	3/58	20/284	7/72	4/187	26/227
%	6.8	8.2	7.2	5.0	9.9	5.2	7.0	9.7	2.1	11.5
***A. lumbricoides***										
Light n/N	69/212	21/59	90/271	49/152	41/119	13/22	50/141	27/108	50/155	40/116
%	32.5	35.6	33.2	32.2	34.5	59.0	35.5	25.0	32.3	34.5
Moderate n/N	111/212	29/59	140/271	74/152	66/119	12/22	92/141	36/108	78/155	62/116
%	52.4	49.2	51.7	48.7	55.5	54.5	65.2	33.3	50.3	53.4
Heavy n/N	32/212	9/59	41/271	29/152	12/119	3/22	31/141	7/108	27/155	14/116
%	15.1	15.3	15.1	19.1	10.1	13.6	21.9	6.5	17.4	12
**Hookworm**										
Light n/N	27/27	46/46	73/73	30/30	43/43	5/5	40/40	28/28	20/20	53/53
%	100.0	100.0	100.0	100.0	100.0	100.0	100.0	100.0	100.0	100.0
Moderate n/N	0/27	0/46	0/73	0/30	0/43	0/5	0/40	0/28	0/20	0/53
%	0.0	0.0	0.0	0.0	0.0	0.0	0.0	0.0	0.0	0.0
Heavy n/N	0/27	0/46	0/73	0/30	0/43	0/5	0/40	0/28	0/20	0/53
%	0.0	0.0	0.0	0.0	0.0	0.0	0.0	0.0	0.0	0.0

^a^Positive students/total students in the group.

^b^Percentage.

Hookworm infection intensity classes were of light infection intensity (100%) independent of municipality, sex, age group and urban/rural schools studied. In the case of *T. trichiura*, light-infection intensity in girls and in the urban schools was higher with statistical differences than in boys (*p* = 0.003) and in the rural schools (*p* = 0.0007). In the case of *A. lumbricoides*, moderate and heavy infection intensities were higher, with statistical differences (*p* < 0.0001 and *p* = 0.003, respectively) in the 6–11-year-old age group.

Table 5 shows the frequency of STH infection grouping. Infection with two STH species is the most frequent association found in R.A.A.N., with the association of *T. trichiura* and *A. lumbricoides* standing out.

**Table 5:** Frequency of STH infection grouping obtained in the two municipalities studied and in the total R.A.A.N. (Nicaragua).

	Puerto Cabezas N = 318		Siuna N = 417		R.A.A.N N = 735		*p* value
	n^a^	%^b^	n	%	n	%	
**STH combination**	295	92.8	158	37.9	453	61.6	0.0001
**1 STH**	96	30.2	91	21.8	187	25.4	0.679
*T. trichiura*	81	25.5	67	16.1	148	20.1	0.130
*A. lumbricoides*	14	4.4	15	3.6	29	3.9	0.999
Hookworm	1	0.3	9	2.2	10	1.4	0.001
**2 STH**	174	54.7	53	12.7	227	30.9	0.0001
*T. trichiura* + *A. lumbricoides*	173	54.4	30	7.2	203	27.6	0.0001
*T. trichiura* + Hookworm	1	0.3	23	5.5	24	3.3	0.0001
**3 STH**	25	7.9	14	3.4	39	5.3	0.023
*T. trichiura* + *A. lumbricoides* + Hookworm	25	7.9	14	3.4	39	5.3	0.023

^a^Positive students in the group.

^b^Percentage.

Only single Hookworm infections appear more frequently in Siuna municipality, with significant differences (*p* = 0.001). However, double and triple STH infections appear more frequently in Puerto Cabezas than in Siuna municipality, with significant differences (*p* < 0.0001 and *p* = 0.023, respectively). Significant positive associations were reached in the co-infection analysis of *T. trichiura* with respect to other STH detected in this study, but no associations were observed between *A. lumbricoides* and Hookworm co-infections.

### Univariable and multivariable analyses of factors related to infections

We assessed the association between infection and several variables to determine their impact on the intestinal parasite infection. During univariable analysis, 3 variables were identified with *p* value < 0.05 in relation to any infection status, such as children’s age, source of drinking water and habit of wearing shoes (Table 6).

**Table 6:** Univariable analysis of variables related to any intestinal parasite infection, any protozoa and any helminth infection among children of R.A.A.N. (Nicaragua).

Variable	Any infection				Any protozoa				Any helminth			
	Positive	Negative	OR	*p* value	Positive	Negative	OR	*p* value	Positive	Negative	OR	*p* value
	n (%)^a^	n (%)	(95% CI^b^)		n (%)	n (%)	(95% CI)		n (%)	n (%)	(95% CI)	
**Gender**												
Female	380 (96.7)	13 (3.3)	1.54 (0.64–3.71)	0.339	357 (90.8)	36 (9.2)	0.97 (0.59–1.61)	0.917	247 (62.8)	146 (37.2)	0.9 (0.67–1.22)	0.498
Male	333 (97.4)	9 (2.6)			310 (90.6)	32 (9.4)			206 (60.2)	136 (39.8)		
**Age group (years)**												
2–5	195 (93.3)	14 (6.7)	5.29 (1.86–15.06)	0.002*	191 (91.4)	18 (8.6)	0.96 (0.53–1.74)	0.901	99 (47.3)	110 (52.6)	2.65 (1.87–3.75)	0.0001*
6–11	398 (99.0)	4 (1.0)			366 (91.0)	36 (9.0)			284 (70.6)	118 (29.4)		
12–15	120 (96.7)	4 (3.2)	2 (0.64–6.28)	0.235	110 (88.7)	14 (11.3)	0.744 (0.36–1.56)	0.432	70 (56.5)	54 (43.5)	1.43 (0.91–2.23)	0.119
**School area**												
Urban	319 (98.2)	6 (1.8)	0.46 (0.18–1.20)	0.112	296 (91.1)	29 (8.9)	0.93 (0.56–1.54)	0.784	224 (68.9)	101 (31.1)	0.57 (0.42–0.77)	0.0001*
Rural	394 (96.1)	16 (3.9)			371 (90.5)	39 (9.5)			229 (55.9)	181 (44.1)		
**Drinking water source**												
Well water	74 (91.4)	7 (8.6)	3.92 (1.51–10.13)	0.005*	73 (90.1)	8 (9.9)	0.93 (0.43–2.03)	0.858	39 (48.1)	42 (51.9)	2.8 (1.74–4.50)	0.0001*
Rain water	538 (97.6)	13 (2.4)			493 (89.5)	58 (10.5)			398 (72.2)	153 (27.8)		
**Open air defecation**												
No	567 (96.6)	20 (3.4)	–	–	529 (90.1)	58 (9.9)	0.49 (0.22–1.11)	0.088	400 (68.1)	187 (31.9)	2.1 (0.96–4.62)	0.063
Yes	44 (100)	0 (0)			36 (81.8)	8 (18.2)			36 (81.8)	8 (18.2)		
**Wearing shoes**												
Yes	18 (90.0)	2 (10.0)	6.04 (1.10–33.35)	0.039*	12 (60.0)	8 (40.0)	1.59 (0.63–4.02)	0.332	15 (75.0)	5 (25.0)	3.51 (1.18–10.51)	0.025*
No	272 (98.2)	5 (1.8)			195 (70.4)	82 (29.6)			253 (91.3)	24 (8.7)		

*Significant association (5% level).

^a^Positive students in the group (percentage).

^b^95% confidence interval.

The odds of helminth infection were highest among 6–11-year-old children, in those drinking rain water (*p* < 0.0001) and in those walking barefoot (*p* = 0.025). On the other hand, the odds of helminth infection were lowest among children of the urban schools (*p* < 0.0001).

The variables with *p* < 0.05 in the univariable analysis were analyzed using multivariable logistic regression model using the backward stepwise method to assess the helminth infection (Table 7). The variable urban/rural school area was left out with *p* value = 0.06. The model identified that the risk of helminth infection is multiplied by 2.6 if the child is 6–11 years old, by 2.9 if the child does not drink safe water, and by 2.5 if the child walks barefoot.

**Table 7:** Multivariable logistic regression analysis of risk factors related to helminth infections in R.A.A.N. (Nicaragua).

Variable	Any helminth	
	OR (95% CI^a^)	*p* value
**Age group (years)**		
2–5	Reference	
6–11	2.55 (1.70–3.81)	0.0001
12–15	1.77 (1.03–3.03)	0.039
**Drinking water source**		
Well water	Reference	
Rain water	2.85 (1.75–4.63)	0.0001
**Wearing shoes**		
Yes	Reference	
No	2.54 (1.12–5.78)	0.025

Factors identified as statistically significant at the 5% level in univariable analysis were entered into a stepwise forward logistic regression model.

^a^95% confidence interval.

## Discussion

The very high infection prevalence of intestinal parasites detected in children from R.A.A.N. is worth mentioning. In fact, it is the highest detected in Nicaragua, with the municipality of Puerto Cabezas standing out. The results obtained are likely to be the consequence of drinking and eating contaminated water and food, habits of playing or handling infested soil, eating with soiled hands, and unhygienic toilet practices. All of this is another clear example of the typical scenario of intestinal parasites present in LAC countries considered climatically suitable for parasite transmission. Besides, the presence of commensal protozoan species is important since they are indicators of poor hygienic-sanitary conditions and that have transmission mechanisms identical to the pathogenic species.

*Blastocystis* spp. and *T. trichiura* were detected as the predominant enteropathogens among children from R.A.A.N., just as in previous Nicaraguan studies^18–21^ and other countries^24–26^. However, in other regions of Nicaragua, *G. intestinalis* and *A. lumbricoides* were the most common parasites found^1,27^.

Focusing on the two studied municipalities, significant differences appear with helminth infection mainly in Puerto Cabezas, while in Siuna protozoan infection is higher. In addition to the scarce knowledge and awareness of parasitic infections^24,28,29^ and the similar hygienic-sanitary conditions in both municipalities, it could be the climatic conditions that mark the differences. Although these two municipalities are separated by only about 210 km, the climate is different between them: Puerto Cabezas presents a tropical rainforest climate, while Siuna is characterized by a tropical monsoon climate. Besides, Puerto Cabezas is a coastal, more urbanized, municipality than Siuna, which is located in the interior and basically rural. This fact indicates that results from one geographical setting might not be applied to other nearby, and thus, climatic conditions, surface temperature and humidity could be more favorable for the survival of one parasite or another.

Each of the infective stages of STH (eggs of *T. trichuris* and *A. lumbricoides*; infective larvae of hookworm) has its climatic thresholds for growth and development. In fact, altered climatic parameters due to climate change are bound to influence the biological development of STH.

The high relative humidity of Puerto Cabezas throughout the entire year could be one of the causes of the higher viability of STH eggs^30^ showing the dominance of *A. lumbricoides* and *T. trichiura*. Both species are more susceptible to increasing temperature, while in the case of Hookworm the trend is contrary and the higher prevalence rates are found in Siuna, more related to the fact that the prevalence of infection is apparently more susceptible to increasing desiccation besides a decreasing level of urbanization^26,31^.

Due to the climatic variability, we found that human helminthiases present a heterogenic distribution in children depending on where exactly they live, even within the same region of Nicaragua (R.A.A.N.). Likewise, significant regional differences in the prevalence and intensity of STH infections have been found in other LAC countries such as Ecuador^38^ and Paraguay^32^.

Similar to other LAC countries^25,32^, no differences of infection between boys and girls were found. However, prevalence of infection was directly related to age, being highest in the 6–11-year-old group, related to starting playing outdoors and getting exposed to infections^26^. Moreover, polyparasitism, the most common form of infection, was also age-related. These results agree with previous work in Nicaragua^27,28^ and other countries^11,34^.

Disease caused by STH is directly related to infection intensity. Although prevalence is used in STH epidemiological surveys, intensity of infection is more important as a determinant of morbidity^35^. The light intensity of *T. trichiura* is normal in endemic areas where most students harbor low or moderate burdens, while only a few individuals harbor a heavy burden^19,33,36^. Moreover, the moderate intensity of *A. lumbricoides* appears mostly in children of 6–11 years of age. In agreement with our results, surveys from other parts of the world also found light intensities of *T. trichiura* and hookworm infections, while values for *A. lumbricoides* were of light and moderate intensities^35^.

Adequate sanitation facilities may be important when explaining the spatial variation of helminth infection prevalence^11^. The odds of STH infection among children of R.A.A.N. who had safe water sources at their disposal were lower compared to those who had to rely on unsafe water sources (rain water). This finding corroborates previous LAC study^37^ and it is in line with the result of a systematic review and meta-analysis which stated that "access to piped water was associated with lower odds *of A. lumbricoides* and *T. trichiura* infection"^38^, as having a water tap means easy access to water for hygienic practices.

The odds of STH infection increase with age since there is an age-related change in exposure to STHs, considering that as they get older they are able to play on their own in outdoor activities. This trend was also observed in other studies conducted elsewhere^39^.

There has been an increase in attention to wearing shoes for preventing soil-related disease conditions as well as STH infection. Previous research estimates that shoe-wearing lowers the odds of Hookworm infection^40,41^. The prevalence of Hookworm infection in our work is not the most commonly STH infection detected, but the lack of shoes is actually being a surrogate marker of lower socioeconomic status, which therefore increases the odds of total STH infection.

Effective strategies for controlling STH infection would have to center on reducing risk factors together with regular deworming, as anthelminthic drugs kill the parasite within the host but do not prevent reinfections and will not be sufficient to bring prevalence to low levels^35,42^. In accordance with WHO guidelines^8^, the children from R.A.A.N. are exposed to a high risk of STH infection (prevalence of any STH > 50%) and should thus continue to be treated with anthelmintics twice a year. Moreover, there is a need to increase people’s knowledge on proper and environmental hygiene, mainly in those communities with lack of water sanitation and hygiene (WASH) such as Puerto Cabezas and Siuna. Results obtained in our work can be used by policy makers in Nicaragua to design geographically more specific intervention programs to reduce STH infections.

Collaborations between research disciplines and between researchers and workers in the field responsible for individual and community health (e.g. parasitology, biochemistry, molecular biology, epidemiology, public health, etc.) is imperative to improve outcomes for people at risk from these STH infections.

The results of our study should be interpreted in the light of some limitations, mainly: only two municipilaties have been studied out of the eight that comprise R.A.A.N.; the used of Kato-Katz technique, which can miss some egg count in an area with light-intensity infections; the prevalence obtained for *S. stercoralis* larvae may not be considered definitive because the specific etiological technique for the detection of this nematode was not applied; and also, the low number of completed questionnaires, as well as the veracity of their responses.

## Data Availability

All the material analyzed as well as the database is available in the Laboratory of Parasitology of the University of Valencia from the corresponding author on reasonable request.
